# Socioeconomic inequalities and mental stress in individual and regional level: a twenty one cities study in China

**DOI:** 10.1186/s12939-015-0152-4

**Published:** 2015-03-07

**Authors:** Hongmei Wang, Xiaozhao Y Yang, Tingzhong Yang, Randall R Cottrell, Lingwei Yu, Xueying Feng, Shuhan Jiang

**Affiliations:** Department of Social Medicine, Zhejiang University School of Medicine, Hangzhou, 310058 China; Department of Sociology, Purdue University, West Lafayette, IN 47907 USA; Public Health Studies Program, School of Health and Applied Human Sciences, University of North Carolina, Wilmington, NC 28403 USA

**Keywords:** Mental stress, Socioeconomic inequalities, China

## Abstract

**Objectives:**

This study will examine explanatory variables including socioeconomic inequalities related to mental stress at both the individual and regional level.

**Methodology:**

A cross-sectional multistage sampling process was used to obtain participants. Data on mental stress and individual socioeconomic status were gathered via face to face interview. Regional variables were retrieved from a national database. Multilevel logistic regression analysis was used to assess socioeconomic variances in mental stress.

**Results:**

Among the 16,866 participants, 27.2% reported severe levels of mental stress (95% CI: 19.4%-35.1%). Multilevel regression analysis indicated that lower individual educational attainment and income, and lower regional Per Capita GDP was associated with mental stress. The results also indicated that managers, clerks, and professional workers manifested higher stress levels than those in other occupations.

**Conclusions:**

Based on the results of this study individual and regional socioeconomic inequalities in China are associated with mental stress.

## Background

Mental stress is a pervasive aspect of life. A certain amount of stress is generally regarded as stimulating and life enhancing, whereas severe and persistent stress may induce poor health, and adversely influence quality of life [1]. Since the late 80’s, China has been transitioning from a centralized to a market-based economy. Massive social change has occurred as a result of this transition [2,3]. The market transition has greatly promoted social economic development and provided improved living standards, and increased choices in consumption, education, health, and employment for the Chinese population [3]. However, the transition has also created many social problems, such as, an imbalance between urban and rural development, regional development differences, rampant corruption, and a widening chasm between the rich and poor. A small minority of individuals in China control most of the resources. Income inequality began in the 1980’s, rose sharply in the 1990s, and has continued to increase in 2000s [3]. People in the bottom levels of society patently do not share social resources equally with those in the top levels of society, and their basic living conditions, including housing, pensions and health care are not guaranteed [3,4].

Studies suggest that people experiencing such unequal conditions endure severe mental stress [1,5]. Stress represents a major social and public health problem in China, with an estimated 173 million Chinese adults having a mental disorder [6]. Stress-related health problems have escalated in China. The World Health Organization (WHO) estimated that neuropsychiatric conditions and suicide together accounted for more than 20% of China’s total illness burden in 2004 [7]. By virtue of its large population, even a conservative estimate indicates that China has the largest number of reported suicides in the world. Globally, Chinese suicides represent 25% to 33% of all documented suicides in the world. At least 600–800 Chinese kill themselves daily [8].

Ecological models have emphasized that mental stress is influenced by both individual and environmental variables. Understanding this relationship is important to control severe mental stress. However, many mental stress studies have examined only the individual correlates [9-11]. Nevertheless, low socioeconomic status (SES) has been associated with high mental stress and psychiatric disorders [11-13]. Given China’s regional differences in SES, it would seem that region of residence might also be related to mental stress, but few studies have directly examined this variable [9,14], direct evidence of regional stress relationships is not available. This is because most studies have been confined to local and community subpopulations, and therefore have had no basis for analyzing regional variation in mental stress [9,10]. Region of residence, however, is a stable estimate of people’s social and economic environments; furthermore, analysis combing individual with regional level data can avoid ecologic and atomistic fallacies, and thus allow for separation of individual and contextual effects on mental stress [15]. China mainland has a vast territory, cultural diversity, and large differences in economic and social development. By utilizing a large-scale, national population sample it is possible to obtain information on the impact of environmental and socioeconomic factors on stress and health.

This study will examine explanatory variables including socioeconomic inequalities related to mental stress at both the individual and regional level. The information from this study could be helpful to inform health policy, plan prevention strategies, and design and implement appropriate, targeted interventions.

## Methods

### Study area and participants

This study employed a cross-sectional multistage sampling design. In Stage 1, twenty-one cities were selected from across China and differentiated by regional location. Nine were located in the east, five in the central region, and the remaining seven in the west. These cities included the more regions of 31 provinces, municipalities and autonomous regions of mainland China. Stage 2 involved selection of residential districts within each city. Two residential districts were randomly selected in the main urban zone of each city, excluding new building districts and subdistricts. In Stage 3, four communities were randomly selected within each residential district. In Stage 4, a family household registration (“hukou”) list was used to randomly sample households in each community. Individuals aged 15 years and older, who were permanent residents, were identified within each household. Finally, one eligible participant was randomly selected from each family, with eligibility being determined by birthdate closest to the contact date to ensure equal representation of the sample [16].

### Data collection

A face-to-face interview was scheduled once an individual was identified and agreed to participate in the study. All interviews were conducted using a structured self-administered questionnaire. Interviewers were fourth-year medical students from a local medical college, who had received one-day training on the study protocol and interview procedures. The survey was administered privately to participants in their home or a designated quiet place, such as a backyard or community park. Interviews were conducted on Saturdays, Sundays, weekday evenings or other times when participants were available. Each participant was asked to fill out a survey questionnaire of approximately 30 minutes duration, following verbal instructions from an interviewer. Each participant had an opportunity to seek information or clarification about the questionnaire items, and were given adequate time for questionnaire completion. Investigators checked returned questionnaires for completeness. Participants were requested to resolve any omissions, as appropriate, and were given a token of appreciation (toothbrush and toothpaste, and other small gifts valued at approximately $ 1.00 US currency) following questionnaire completion.

A common research protocol was utilized across all 21 cities to assure homogeneity of interview and data collection. The study was approved by the Ethics Committee at the Medical Center, Zhejiang University, and verbal consent was obtained from all participants prior to data collection. Our methods have been extensively employed in smoking research in China, and they possess acceptable validity [5,11].

### Measures

#### Dependent variable

Mental stress status: Mental stress was measured by the Chinese version of the Perceived Stress Scale (CPSS) [5,11,17]. This scale comprised 14 items that addressed perceptions of stress during the month prior to the survey. Items were rated on a 5-point Likert-type scale and ranged from 0 (never) to 4 (very often). Item scores were summed to yield a total stress score, with higher scores indicating higher perceived levels of stress. This scale has been widely used to assess stress in China and has been shown to be an appropriate indicator of mental health status [11,18,19]. Following previous practice, severe stress was operationalized as a score >25, which was classified by ROC (Receiver Operating Characteristic Curve) performance using mental disorders gold standard. This classification has demonstrated acceptable sensitivity and specificity [5]. The dependent variable in this study was severe mental stress (SMS), and was coded dichotomously as 1 = no severe mental stress and 2 = severe mental stress.

#### Individual-level independent variables

Sociodemographic questions to determine such variables as age, gender, ethnicity, educational level, occupation, and income were included on the questionnaire.

#### City -level independent variable

There were six independent variables that reflected potential regional variation in the characteristics of the 21 study cities. The first was regional location, distinguished as east, central, and west. The second aspect was population size, which categorized these cities as being less than 5 million, from 5 million to less than 10 million, 10 million and more population. The third aspect was level of economic development (per capita Gross Domestic Product (GDP) in yuan), which was categorized as less than 40,000, from 40,000 to less than 60,000, 60,000 and more. The above data were obtained from the National Bureau of statistics [20].

### Data analysis

All data were entered into a database using Microsoft Excel. The dataset was then imported into SAS (9.3 version) for statistical analyses. Descriptive statistics were calculated for SMS prevalence. Chi-square analyses were used to determine differences in SMS across the selected cities using SAS 9.3 survey procedure (SAS institute Inc, 2001). Associations were confirmed through application of a multilevel logistic regression model using the SAS NLMIXED procedure [15].

Two models in the multilevel analysis. First the Null Model, a two-level model with random intercepts, was developed. It did not include any predictors except a constant, in accounting for variation in SMS across our twenty one study cities [21,22]. In this base model, all demographic and regional level variables were entered as fixed main effects to form the full model to evaluate the separate impact of all individual-level and regional-level variables on SMS [21,22]. For this analysis, the dependent variable, SMS, was operationalized as a binary response (1 = no severe mental stress and 2 = severe mental stress). The independent variables in this analysis were those emerging as statistically significant (p < 0.1) in the Chi-square tests. These results are presented in Table 1. The first category in each variable served as the referent in the multilevel logistic regression analysis. Model fitting was assessed by the likelihood of a change in the −2 log. Significance of the random parameter variance estimates was assessed using the Wald joint *X*^2^ test statistic [21,22].

**Table 1:** **Demographic characteristics of the sample and SMS prevalence**

**Group**	**N**	**% of sample**	**Prevalence (%)**	**Rao-Scott Chi-Square**	**P**
Age (years)				0.15	0.9977
<25	3010	15.0	26.4		
25-34	4394	20.7	25.9		
35-44	3883	19.7	25.5		
45-54	2755	19.5	27.3		
55+	2824	25.0	26.2		
***Ethnicity***				0.26	0.6098
Han	15203	91.9	26.5		
Other	1663	8.1	22.7		
Gender				6.83	0.0089
Male	8795	46.9	32.8		
Female	8071	53.1	20.4		
***Education***				12.97	0.0047
Elementary school or less	1899	10.7	48.4		
Junior high school	5213	27.0	29.8		
High school	4914	31.0	19.1.		
Junior college or more	4840	11.3	22.6		
***Occupation***				26.07	0.0002
Managers and clerks	1553	7.5	61.7		
Professionals	1291	6.2	40.5		
Commerce and service	2914	15.7	26.9		
Operations	2344	23.8	22.4		
Students	1541	8.1	29.5		
Retired	2286	21.1	17.4		
Other	4932	28.6	20.7		
***Income/person/year*** Yuan*				111.87	<0.0001
<10,000	3818	21.6	43.4		
10,000-19,999	5090	27.2	33.3		
20,000-29,999	3677	21.2	18.8		
30,000-39,999	1754	12.2	10.8		
40,000-49,999	1157	8.2	11.4		
50,000+	1370	9.8	16.4		
**Region variance**					
***Region location***				0.73	0.6939
East	6469	49.8	23.1		
Central	2566	10.6	26.2		
West	7831	39.6	30.3		
GDP				16.25	0.0003
<40,000	7713	39.0	36.1		
40,000-59,999	3944	26.1	30.4		
60,000+	5209	34.8	12.1		
***Population(million)***				7.20	0.0275
<5	7026	22.0	23.6		
5-	5960	30.8	28.4		
10 -	3880	46.9	16.6		

*1Chinese yuan = $6.5.

All analysis were weighted [15]. Weights included (1) sampling weights as the inverse of the probability of selection at each level including regional, city, district, and community levels, and they were multiplied together for each level. (2) The non-participation weight covered two aspects. Household-level non-participation weights were calculated at the city level. Individual-level non-participation weights were based on a combination of city, age, and sex, and the corresponding weight was the inverse of the participation rate. The overall non-participation weights were computed as the product of the household and individual weights (3) post-stratification weights were made by the combination of sex (male, female) and age (<25 year, 25–34, 35–44, 45–54, and 55+) based on estimated distributions of these characteristics from a national survey [23]. The final overall weights were computed as the product of the above three weights [15,16].

The above process is visually depicted using the below flow chart.



Chi-square analyses were weighted using the overall participant-level weights, and the multilevel analysis was weighted using sampling weight in city level and subject-level weights with non-participation and post-stratification weights, respectively [15]. As there is no weight statement available for the NLMIXED procedure, these analyses were weighted though a macro method in this study [15].

## Results

A total of 18,875 individuals were identified as potential subjects for this study, among whom, 17,124 were effectively contacted and agreed to participate in the survey. Of the 17,124 surveys, 16,866 (96.8%) were fully completed, valid questionnaires and utilized in this study.

The average stress score for the 16,866 respondents was 22.47 (95%CI: 21.32, 23.63), the median stress score was 24.1651 (0–56), and 26.21% (95%CI: 16.8%, 35.7%) of participants were categorized as SMS (25 or above stress score). Our univariate data indicated that resident sex, education attainment, occupation, and income were associated with SMS (Table 1). Regionally, population and Per Capita GDP were also associated with the SMS. Table 2 shows parameter estimates in multiple level models. Random and fixed parameters were statisticly significantly in the Null model. Random parameter declined, and fixed parameter increased but significance level declined from the null model to the full model.

**Table 2:** **Rarameter estimates from multiple level models**

**Group**	**Null model**	**Full model**
Random parameters between regions	1.3006(0.3622)**	0.9596(0.3146)**
Fixed parameters	0.9565(0.3146)**	1.1165(0.4931)*

*p < 0.05.

**p < 0.01.

Table 3 presents results of the multilevel logistic regression model analyses of SMS. In the null model, the random coefficient estimates indicated significant inter-regional variation in stress (P < 0.01). The full model indicated that those with elementary school or less education levels had a higher stress level than those with high school, junior college or college education levels. Considering occupational groups, managers, clerks, and professional workers reported higher stress levels than those in other occupational groups. The residents earning higher annual incomes appeared less stressed than those earning lower annual incomes. At the regional level residents with higher Per Capita GDP manifested less mental stress than those with lower GDP. There was no interactions found between individual income and regional GDP (Estimate: 3188, SE:0.1713, p: 0.0628) in the full model.

**Table 3:** **Results of multiple level analyses**

**Group**	**OR**	**95%C.I**
**Individual level**		
***Education***		
Elementary school or less	1.00	
Junior high school	0.62	0.38,1.03
High school	0.37	0.18,0.78**
Junior college or college	0.48	0.24,0.93*
***Occupation***		
Managers and clerks	1.00	
Professionals	0.52	0.29,1.02
Commerce and service	0.24	0.10,0.62**
Operations	0.27	0.11,0.67**
Students	0.22	0.07,0.70**
Retired	0.24	0.12,0.50**
Other	0.27	0.12,0.59**
***Income/person/year*** Yuan*		
<10,000	1.00	
10,000-19,999	0.82	0.59,1.13
20,000-29,999	0.42	0.33,0.54**
30,000-39,999	0.21	0.12,0.39**
40,000-49,999	0.21	0.11,0.42**
50,000+	0.27	0.17,0.43**
***Regional level***		
Per Capita GDP(yuan)		
<40,000	1.00	
40,000-59,999	0.95	0.44,2.21
60,000+	0.28	0.12,0.65**

*p < 0.05.

**p < 0.01.

## Discussion

Based on the results of this study, the average stress score for all respondents was 22.47 (95%CI: 21.32, 23.63), which was lower than the mean score of 23.90 found in the 2001 year survey (95%CI: 23.68, 24.12), but not significantly different from the mean of 23.69 (95%CI: 23.38, 24.10) in the 2008 survey [11]. Nevertheless, mean stress levels and high stress levels are higher in China than in most other countries.

There may be numerous explanations for China’s high stress levels. China has been transitioning from a centrally-planned/controlled to a market-oriented economy. In undergoing this change it has passed through four stages. The first stage was the preparation period (1978–1987), the second stage the moderate period (1988–1994), and the third stage the radical period (1995–1999). The fourth and current stage, commencing in 2000, is known as the regulation period. Many social issues, which had manifested in the moderate period, intensified in the radical period. The radical period saw abolition or deregulation of numerous state-owned enterprises and of the loss of many low level, menial jobs [3,5,6]. Those working in these low level jobs have few alternatives when their jobs are eliminated and there are fewer overall jobs at this level. During the radical period, serious inequalities emerged. Disproportional sharing of financial resources induced vast income inequality among the various economic sectors and regions of China. This profound change from a period when resources were more equally distributed may contribute to high stress levels among urban residents [3]. At the same time, modern lifestyles, with their strong social competition and time demands, may also elevate stress levels [5]. In the current period, the Central Government to some extent been cognizant of these problems and begun to take preliminary steps to protect disadvantaged groups. However, the mental stress levels is still high among residents, which is a clear indicator that social inequality problems have not been completely resolved. Many studies have reported that severe and enduring stress may induce poor health, and adversely influence quality of life [1,5,24]. This situation likely harbors important implications for underlying physical disease and psychiatric disorders, and reveals a need for government officials to consider disease prevention and health promotion as part of stress-reduction initiatives.

Socioeconomic inequalities are generally associated with stress and mental disorders. This study provided additional evidence that lower education attainment and income are associated with higher mental stress, which is consistent with prior Chinese studies [5,17]. It has been suggested that those with lower education levels have less recourses and opportunities, and probably have fewer coping skills for confronting life challenges [11,13,24]. The association between urban residents with lower incomes and higher stress levels has also been found in other cultures [22,25]. Higher economic attainment means better educational opportunities, more extensive social networks, more personal freedom, and healthier and safer work environments [24-27].

In this study stress also varied by occupation. Managers, clerks, and professionals were more stressed than others. The association between occupation and mental stress is a very complex issue. It is suspected that people's occupational status directivity reflects their socio-economic status much like the relationship between education and income. Managers, clerks, and professionals should enjoy a higher socioeconomic position in Chinese society; yet, they have high stress levels. This might be due to job functions [25]. Though managers and clerks have relatively stable socioeconomic status, these employees also have highly demanding, competitive jobs. Many are employed in government organizations that have had the most “institutional reform” in recent years [2,4,11]. Consistent with prior studies professionals also reported high stress levels [11], and this might be related to the increased social competition for such jobs.

This is the first study to document an association between geographic region, socioeconomic inequities and mental stress. This is an important finding as socioeconomic variables are stable, and relate to all groups within the region. Residents located in lower regional Per Capita GDT regions have higher SMS prevalence, which strengthens the association between income and mental stress.

Whether individual education and income or regional Per Capita GDP reflects socioeconomic status, this study supports the notion that socioeconomic inequities contribute to SMS. The stress theory postulates that personal resources, such as coping style, self-esteem, mastery, and locus of control, buffer the impact of stress on depression and that higher-SES individuals are better equipped with such resources [28]. The social ecological theory addresses the impact of regional features such as socioeconomic status, social cohesion, infrastructure, and public health policy on health [12,25]. Results from this study are consistent with these theories.

The results of this study strongly suggest the importance of combating persistent and relatively high stress levels among residents. The Central Government and local health authorities need to collaborate on policies for stress reduction and the prevention of mental disorders. Socioeconomic inequities must be reduced, and benefits made available to the large disadvantaged element of society [29]. Prevention needs to target those urban residents who are at risk of severe stress due to low socioeconomic status. A nationwide media campaign should be planned and implemented to educate the populace about the adverse health effects of stress. Support groups should be established in urban community centers to provide a forum where residents can express mental health concerns, talk with others having similar concerns, and learn stress-reduction and management skills. Worksite programs should be established to help employees manage their stress at work and at home. Local health authorities should offer stress management programs and concurrently offer mental health treatment as needed. Special community-based clinics should be established to provide high-risk individuals with no or low cost psychological counseling on an as-needed basis and in-patient care if required.

This study has two limitations. First, this is a cross-sectional study, which precludes causal inference. However, this study is large-scaled and results can provide some suggestions regarding the important associations between socioeconomic status and high stress levels. Second, the sampling frame focuses on urban areas of China and included only 21 large cities. Hence, findings of the study cannot be generalized to all of China and especially not to those Chinese living in rural areas.

## Conclusion

The association of current socioeconomic inequalities and mental stress holds important implications for mental health services in China. Our findings provide evidence to inform policymakers, and strongly encourage them to develop and implement effective policies and interventions.

## Appendix

### The project collabration group

Local collaborating and PI

Angui Medical University (Yang Jinxia), Baotou Medical College (An Jinagang), Chengdu Medical College (Cheng Jian), Chongqing Medical University (Miao Qing), Dalian Medical University (Li Xiaofeng), Guangxi Medical University (Cheng Zhiping),Guiyang Medical College (Yan Zheng), Hainan Medical College (Yang Jianjun), Hebei Medical University (Tan Fengzhu), Huazhong University of Science and Technology (Du Kaiyu), Jilin University (Li Jinhua), Kunming Medical College (Cui Wenlong), Lanzhou University (Bai Yanai), Nanhua University (Long Dingxin), Nanchang University (Zhou Xiaojun), Qinghai University (Mao Huiqing), Shandong University (Li Jie), Shanghai Jiaotong University (Bao yong), Tian jin Medical University (Ma Jun), Xi An Medical College (Cao Ping), Xinjiang Medical University (Liu Jiwen), Xuzhou Medical college (Lu Zhaojun), Xiamen University (Fang Ya), Zhengzhou University (Fen Qingyun).

## References

[1] Charney DS, Manji HK (2004). Life stress, genes, and depression: multiple pathways lead to increased risk and new opportunities for intervention. Sci STKE.

[2] Hao C (2006). Development of financial intermediation and economic growth: The Chinese experience. China Econ Rev.

[3] Zhou XG (2000). Economic transformation and income inequality in urban China: evidence from panel data. Am J Sociol.

[4] Meng X (2004). Economic restructuring and income inequality in urban China. Rev Income Wealth.

[5] Yang T, Huang HT (2003). An epidemiological study on stress among urban residents in social transition period. Chin Epidemiol.

[6] Phillips MR, Zhang J, Shi Q, Song Z, Ding Z, Pang S (2009). Prevalence, treatment, and associated disability of mental disorders in four provinces in China during 2001–05: an epidemiological survey. Lancet.

[7] World Health Organization: Department of Measurement and Health Information. Death and DALY estimates for 2004 by cause for WHO Member States. Geneva: World Health Organization; 2009. http://www.who.int/healthinfo/bod/en/index.html. Accessed: 10-26-2014.

[8] Lee S, Kleinman A, Perry EJ, Seldon M (2000). Suicide as resistance in Chinese society. Chinese society: Change, conflict and resistance.

[9] Lorant V, Deliège D, Eaton W, Robert A, Philippot P, Ansseau M (2003). Socioeconomic inequalities in depression: a meta-analysis. Am J Epidemiol.

[10] Kristenson M, Eriksen HR, Sluiter JK, Starke D, Ursin H (2004). Psychobiological mechanisms of socioeconomic differences in health. Soc Sci Med.

[11] Yang T, Wu D, Zhang W, Cottrell RR, Rockett IR (2012). Comparative stress levels among residents in three Chinese provincial capitals, 2001 and 2008. PLoS One.

[12] Robert S, House J, Albrecht GL, Fitzpatrick R, Scrimshaw S (2000). Socioeconomic inequalities in health: integrating individual, community and societal level theory and research. The handbook of social studies in health and medicine.

[13] Turner RJ, Lioyd DA (1999). The stress process and the social distribution of depression. J Health Soc Behav.

[14] Weich S, Lewis G, Jenkins SP (2001). Income inequality and the prevalence of common mental disorders in Britain. Br J Psychiatry.

[15] Grilli L, Pratesi M (2004). Weighted estimation in multilevel ordinal and binary models in the presence of informative sampling designs. Surv Methodol.

[16] Yang T, Mao A, Feng X, Jiang S, Wu D, Bottorff J (2014). Smoking cessation in an urban population in China. Am J Health Behav.

[17] Cohen S, Kamarack T, Mermelstein R (1983). A global measure of perceived stress. J Health Soc Behav.

[18] Wang J, Xie W, Li L, Zhou W (2008). Correlations of mental stress, coping style and social support among heroin dependence patients. Chin J Drug Depend.

[19] Li L, Feng L, Wu L, Yang Y (2001). Association between stress and health in radiology physicians. J Environ Occup Med.

[20] Department of Comprehensive Statistics of National Bureau of Statistics (2012). China Statistical Yearbook for Regional Economy 2011.

[21] Goldstein H (1995). Multilevel statistical models.

[22] Wang J, Xie H, Jiang B (2008). Multilevel models :Methods and application.

[23] National Bureau of statistics (2011). China statistical yearbook.

[24] Paul K, Boutain D, Agnew K, Thomas J, Hitti J (2008). The relationship between racial identity, income, stress and C-reactive protein among parous women: implications for preterm birth disparity research. J Natl Med Assoc.

[25] Markwick A, Ansari Z, Sullivan M, Parsons L, McNeil J (2014). Inequalities in the social determinants of health of Aboriginal and Torres Strait Islander People: a cross-sectional population-based study in the Australian state of Victoria. Int J Equity Health.

[26] Ritter C, Hobfoll SE, Lavin J, Cameron RP, Hulsizer MR (2000). Stress, psychosocial resources, and depressive symptomatology during pregnancy in low-income, inner-city women. Health Psychol.

[27] Cottrell RR, McKenzie JF (2011). Health Promotion & Education Research Methods.

[28] Krieger N, Williams DR, Moss NE (1997). Measuring social class in US public health research: concepts, methodologies, and guidelines. Annu Rev Public Health.

[29] Asada Y, Whipp A, Kindig D, Billard B, Rudolph B (2014). Inequalities in multiple health outcomes by education, sex, and race in 93 US counties: Why we should measure them all. Int J Equity Health.

